# Impact of systemic lupus erythematosus on the 5-year survival of critically ill septic patients

**DOI:** 10.1186/s13075-021-02649-x

**Published:** 2021-10-21

**Authors:** Hsin-Hua Chen, Hsian-Min Chen, Yi-Ming Chen, Yi-Hsing Chen, Ching-Heng Lin, Wen-Cheng Chao

**Affiliations:** 1 Division of Allergy, Immunology and Rheumatology, Department of Internal Medicine, Taichung Veterans General Hospital, Taichung, Taiwan; 2 Division of General Internal Medicine, Department of Internal Medicine, Taichung Veterans General Hospital, Taichung, Taiwan; 3 Institute of Biomedical Science and Rong Hsing Research Centre for Translational Medicine, Chung Hsing University, Taichung, Taiwan; 4 School of Medicine, National Yang Ming Chiao Tung University, Taipei, Taiwan; 5 Department of Industrial Engineering and Enterprise Information, Tunghai University, Taichung, Taiwan; 6 Big Data Center, Chung Hsing University, Taichung, Taiwan; 7 Department of Medical Research, Taichung Veterans General Hospital, Taichung, Taiwan; 8 Department of Computer Science & Information Engineering, National United University, Miaoli, Taiwan; 9 Department of Biomedical Engineering, HungKuang University, Taichung, Taiwan; 10 Center for Quantitative Imaging in Medicine (CQUIM), Department of Medical Research, Taichung Veterans General Hospital, Taichung, Taiwan; 11 Department of Healthcare Management, National Taipei University of Nursing and Health Sciences, Taipei, Taiwan; 12 Department of Public Health, College of Medicine, Fu Jen Catholic University, New Taipei, Taiwan; 13 Department of Critical Care Medicine, Taichung Veterans General Hospital, 40705 Taiwan Avenue, Xitun District, No. 1650, Section 4, Taichung, Taiwan; 14 Department of Computer Science, Tunghai University, Taichung, Taiwan; 15 Department of Automatic Control Engineering, Feng Chia University, Taichung, Taiwan

**Keywords:** Systemic lupus erythematosus, Sepsis, Long-term outcome, Mortality, Risk factor

## Abstract

**Background:**

Infectious disease is an increasing threat to patients with systemic lupus erythematosus (SLE); however, the long-term outcome in critically ill septic patients with SLE remains unclear, and we aimed to address the impact of SLE on 5-year survival in critically ill septic patients.

**Methods:**

We used the 2003–2017 nationwide data with 825,556 patients with sepsis in Taiwan. We identified lupus cases with sepsis that required admission to the intensive care unit and mechanical ventilation and selected controls matched (1:4) for age, sex, and index-year. Conditional logistic regression analysis was used to determine risk factors for mortality risk and shown as odds ratios (HRs) with 95% confidence intervals (CIs).

**Results:**

A total of 513 SLE-sepsis patients and 2052 matched non-SLE septic individuals were enrolled. The mortality rate was higher in the SLE group (38.5 per 100,000 person-year) than that in the non-SLE group (13.7 per 100,000 person-year), with an IRR of 2.8 (95% CI, 2.5–3.2). We found that SLE was independently associated with a high mortality rate after adjusting relevant variables (HR 1.47, 95% CI 1.27–1.77). In addition to SLE, a higher age (HR 1.02, 95% CI 1.02–1.02), more comorbidities, and receiving prednisolone equivalent dose higher than 5 mg/day (HR 1.55, 95% CI 1.27–1.90), methotrexate (HR 2.19, 95% CI 1.61–2.99), and immunosuppressants (HR 1.45, 95% CI 1.22–1.74) were also independent risks for mortality.

**Conclusions:**

We identified that SLE affects the long-term mortality in critically ill septic patients, and more studies are warranted for the underlying mechanism.

**Supplementary Information:**

The online version contains supplementary material available at 10.1186/s13075-021-02649-x.

## Background

Systemic lupus erythematosus (SLE) leads to substantial morbidity and mortality due to systemic involvement and adverse effects of medications despite the improved management for SLE in the past four decades [1]. One recent Canadian population-based study has shown the current all-cause age-specific standardised mortality ratio (SMR) of SLE was 2.2 (95% CI 1.4–3.1) compared with those in the general population, and the infectious disease accounts for the majority of mortality, particularly among younger patients with SLE [2]. Furthermore, two population-based studies in the USA and Hungary found a gradual increase of hospitalised infection and sepsis in patients with SLE [3, 4]. Although a number of studies have explored the short-term outcome of lupus patients admitted for sepsis [5–7], however, the distinct impact of SLE on long-term outcome among patients with sepsis remains unclear mainly due to the lack of comparable non-SLE controls [8].

Recently, increasing evidence including our previous studies focusing on critically ill cancer patients have shown that the prolonged sequelae of critical illness may affect the long-term outcome in patients with sepsis [9, 10]. Notably, the altered immunological and metabolic response in the recovery from sepsis may further lead to the vulnerability for secondary infection and systemic diseases including cardiovascular events [11, 12]. Therefore, the long-term outcome, instead of ICU/hospital-mortality, of critically ill patients is currently one of the leading research priorities in critical care medicine, particularly among those with sepsis, given the increasing evidence have shown the prolonged sequelae of sepsis [10, 13, 14]. These evidence highlight the essential needs to address the complex association among SLE, sepsis, and long-term mortality. We hence used a population-based database and case-control design to explore the 5-year mortality in critically ill septic patients, to investigate the impact of SLE on 5-year survival, and to identify factors associated with mortality in critically ill septic patients.

## Materials and methods

### Ethical statements

This study was approved by the Institutional Review Board of Taichung Veterans General Hospital (IRB number: CE19038A). All the individual data were anonymised before analysis, and informed consent was waived.

### Study design and data source

The claim data were obtained from the National Health Insurance Database (NHID) in Taiwan. The National Health Research Institutes (NHRI) maintained all of the enrolment files and original reimbursement claims data obtained from the National Health Insurance (NHI) administration and then released the data to the NHID. The NHID has stored medical claims since 1997 with nearly 99.6% coverage of the 23.3 million Taiwanese residents given that NHI is the compulsory population-based insurance in Taiwan. The medical diagnoses in NHID are based on the International Classification of Diseases, Ninth Revision, Clinical Modification (ICD-9-CM) and ICU-10-CM.

### Definitions of sepsis

In the present study, we used the Sepsis-3 definition, which used the sequential organ failure assessment (SOFA) score as diagnostic criteria, to identify patients with sepsis in accordance with previous studies including our recently published study [15–17]. In brief, the septic episode was defined by a diagnosis of infectious disease and at least one acute organ dysfunction [16, 17]. The definition of acute organ dysfunction consisted of items in sequential organ failure assessment (SOFA) score, including dysfunction of the cardiovascular, respiratory, hepatic, hematologic, renal, and central nervous system. The index-date of sepsis was defined as the first day of an emergency department or hospital visit for sepsis.

### Identification of critically ill septic patients with SLE and matched non-SLE controls in the whole Taiwanese population

**Fig. 1:**
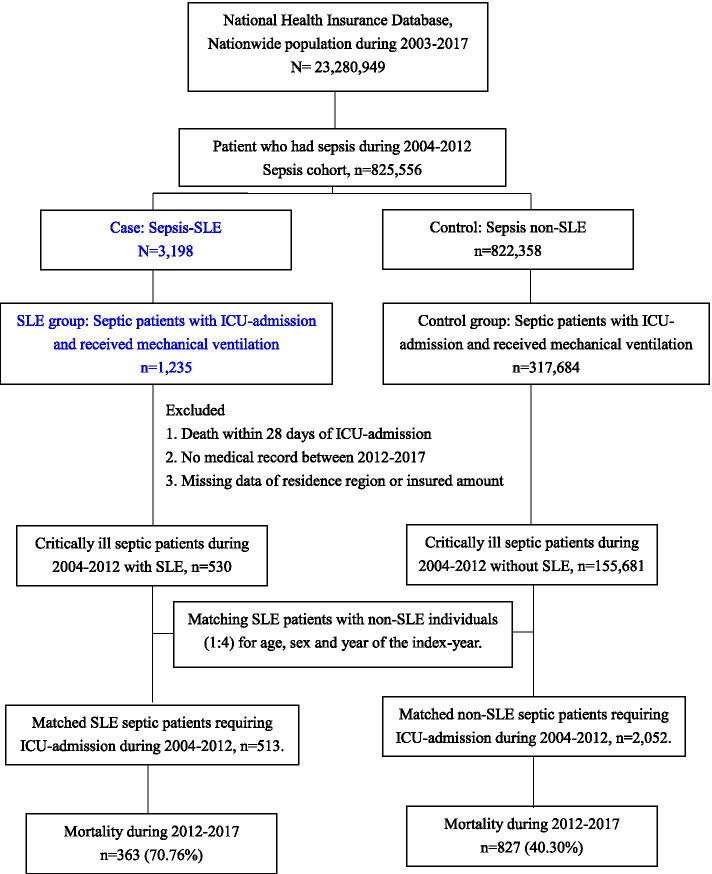
Flow chart of the study design

Given that we aimed to explore 5-year survival using NHIRD 2003-2017, we hence enrolled patients who met the aforementioned criteria for sepsis during 2004–2014. To avoid the inclusion of patients with non-critical sepsis, we applied the stringent criteria with ICU-admission and receiving mechanical ventilation (ICD code, 57001B and 47031C) to define critically ill septic patients. Furthermore, we excluded those who die within 28 days of the index-date of sepsis given that we focused on the prolonged impact of SLE on the long-term mortality in critically ill septic patients. Patients with SLE were defined as having at least three ambulatory visits or one hospital admission with a diagnosis of SLE (ICD code, 710.2 and M32.10) and a catastrophic illness certificate for SLE. In Taiwan, the certificate of catastrophic illness for SLE was issued after reviewing comprehensive clinical data by two rheumatologists, and the copayment was exempted with the certificate. Among critically ill septic patients without SLE, we randomly selected non-SLE septic controls, matching SLE septic cases (1:4) for age, gender, and the index-year (Fig. 1).

### Outcome

In the present study, the primary outcome is mortality, and we linked the death registration database to ascertain the date of death. The censored date was defined as the time of withdrawal from the NHI for any reason, including death or leaving from Taiwan, or 31 December 2017 (the last date of the data used), whichever came first.

### Potential confounders

Potential confounders for adjustment in the conditional Cox regression model included age, gender, Charlson comorbidity index (CCI), and SLE-associated medications. The presence of comorbidity was defined as having at least three ambulatory visits or one inpatient visit with a corresponding ICD-9/10-CM code within 1 year prior to the index-date. We adjusted recent hospitalised infection given that our previous study had shown that hospitalised infection within 3 months was associated with sepsis risk in patients receiving tumour necrosis factor inhibitors for immune-mediated inflammatory diseases [17]. Furthermore, socioeconomic status has been implicated with sepsis, and we hence adjusted the payroll-related insured amount and urbanisation level in this study [17]. In brief, the urbanisation level of the patient’s residence was classified into three clusters based on population density (people/km^2^), population ratio of agricultural workers, population ratio of subjects with educational levels of college or above, population ratio of elderly subjects aged > 65 years, and number of physicians/100,000 subjects [18]. The adjusted medications within one year prior to the index-date consisted of glucocorticoid, methotrexate, sulfasalazine, and hydroxychloroquine as well as immunosuppressants, including cyclophosphamide, azathioprine, cyclosporine, mycophenolate, and mycophenolic.

### Statistical analysis

Descriptive results were presented as means ± standard deviation or number (percentages). The mortality rate was presented as per 100,000 person-years, and the incidence rate ratio (IRR) was calculated. Kaplan–Meier method was applied to compare the cumulative survival in critically ill septic patients with and without SLE. Variables were included in the multivariable model if the associated univariable *p* value was < 0.20 and the variance inflation factor was < 10 [19]. A conditional Cox regression was conducted to estimate the hazard ratio (HR) and 95% confidence interval (CI) of mortality after adjustment for age, gender, CCI, and medications. All the data were analysed using statistical software version 9.3 (SAS Institute, Inc., Cary, NC, USA). A *p* value < 0.05 was considered as statistically significant.

## Results

### Characteristics of enrolled subjects

A total of 513 SLE-sepsis patients and 2052 matched non-SLE septic individuals were enrolled for analyses (Fig. 1 and Supplemental Table 1). We found that SLE patients had a more CCI (2.9 ± 1.7 vs. 2.4 ± 2.6, *p* < 0.001) and were more likely to live in an urbanised area (*p* < 0.001) and were less likely to have a low insured income (47.6% vs. 55.6%, *p* < 0.001). With regard to medications, patients with SLE were more likely to receive glucocorticoid (98.6% vs. 78.3%, *p* < 0.001), methotrexate (3.9% vs. 2.2%, *p* = 0.032), sulfasalazine (1.9% vs. 0.7%, *p* = 0.014), hydroxychloroquine (64.5% vs. 1.8%, *p* < 0.001), and immunosuppressants (57.3% vs. 4.8%, *p* < 0.001) than those in non-SLE critically ill septic patients (Table 1) (see detailed comorbidities in Supplemental Table 2).

**Table 1:** Demographic data and clinical characteristics among septic patients with and without SLE

	SLE	Non-SLE	***p*** value
	(***n*** = 513)	(***n*** = 2052)	
**Age, years**	48.8 ± 16.3	48.8 ± 16.3	1
**Gender**			1
Female	442 (86.2)	1768 (86.2)	
Male	71 (13.8)	284 (13.8)	
**Urbanisation levels**			< 0.001
Urban	164 (32.0)	475 (23.1)	
Suburban	221 (43.1)	947 (46.2)	
Rural	128 (25.0)	630 (30.7)	
**Low insured income**^a^	243 (47.4)	1141 (55.6)	< 0.001
**CCI without renal disease,** mean ± SD	2.9 ± 1.7	2.4 ± 2.6	< 0.001
**CCI without renal disease,** group			< 0.001
0	4 (0.8)	608 (29.6)	
1–3	359 (70.0)	930 (45.3)	
> 3	150 (29.2)	514 (25.0)	
**Recent hospitalised infection**^b^	78 (15.2)	199 (9.7)	< 0.001
**Medications**			
**Glucocorticoid use**	506 (98.6)	1606 (78.3)	< 0.001
**Glucocorticoid dosage, mg/day**^c^	47.9 ± 85.1	15.7 ± 40.2	< 0.001
**Glucocorticoid dosage group**^c^			< 0.001
0 mg/day	7 (1.4)	447 (21.8)	
0-5 mg/day	27 (5.3)	678 (33.0)	
≥ 5 mg/day	479 (93.4)	927 (45.2)	
**DMARD**			
Methotrexate	20 (3.9)	45 (2.2)	0.032
Sulfasalazine	10 (1.9)	14 (0.7)	0.014
Hydroxychloroquine	331 (64.5)	36 (1.8)	< 0.001
Immunosuppressants^d^	294 (57.3)	98 (4.8)	< 0.001

^a^Insured income lower than median income (21,900 New Taiwan dollars). ^b^Within 3 months prior to index admission. ^c^Prednisolone equivalent. ^d^Cyclophosphamide, azathioprine, cyclosporine, mycophenolate, and mycophenolic. *Abbreviations*: *SLE* Systemic lupus erythematosus, *CCI* Charlson comorbidity index, *DMARD* Disease-modifying antirheumatic drug

### Long-term survival in critically ill septic patients with and without SLE

**Fig. 2:**
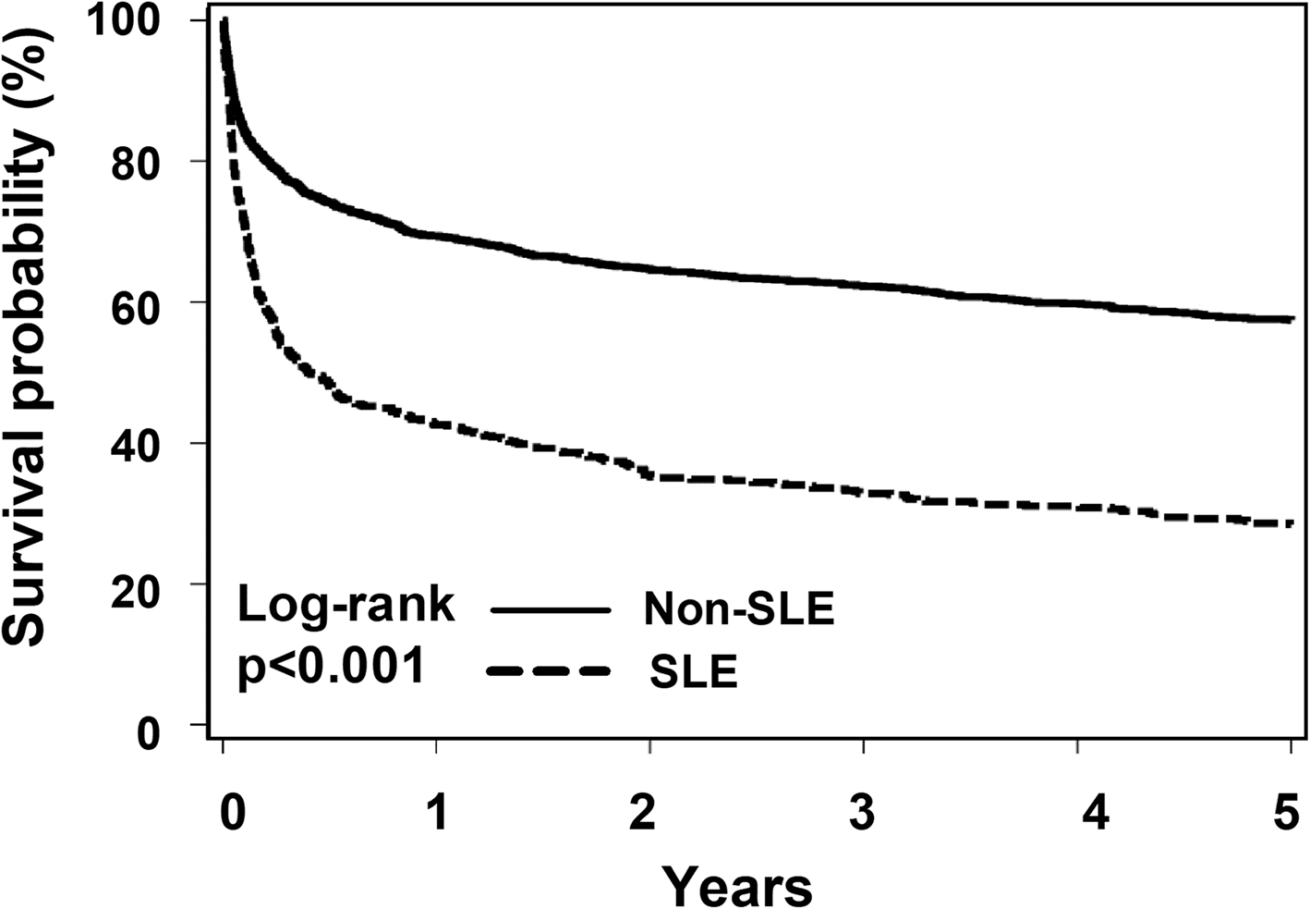
The cumulative survival of critically ill septic patients with and without systemic lupus erythematosus (SLE)

Table 2 shows a comparison of the mortality rate among SLE septic patients with that among non-SLE septic individuals. We found a high proportion of SLE septic patients died within 5 years (70.8%, 363/513), and the 5-year mortality in non-SLE septic patients was approximately 40% (827/2052). The incident mortality rate was higher in the SLE group (38.5 per 100,000 person-year) than that in the non-SLE group (13.7 per 100,000 person-year), with an IRR of 2.8 (95% CI, 2.5–3.2) (Table 2). We further used Kaplan–Meier estimates to illustrate the impact of SLE on the long-term mortality among critically ill septic patients and found a marked increased incidence of mortality among SLE patients, particularly in the first year after being survived from critically ill sepsis (Fig. 2).

**Table 2:** Mortality in patients who survived from sepsis with and without SLE

	Total	Event (%)	Total person-years	Incidence rate (/10^**5**^ years)	Crude IRR (95%CI)
**6-month mortality**					
**SLE**	513	264 (51.5%)	154	171,287	2.62 (2.3–3.0)
**Non-SLE**	2052	517 (25.2%)	792	65,313	1
**1-year mortality**					
**SLE**	513	292 (56.9%)	267	109,410	2.66 (2.3–3.1)
**Non-SLE**	2052	608 (29.6%)	1476	41,188	1
**5-year mortality**					
**SLE**	513	363 (70.8%)	942	38,516	2.8 (2.5–3.2)
**Non-SLE**	2052	827 (40.3%)	6045	13,680	1

*Abbreviations*: *SLE* Systemic lupus erythematosus, *IRR* Incidence rate ratio, *CI* Confidence interval

### Factors associated with risks for mortality in critically ill septic patients

We then estimated the risk for mortality in the 2565 critically ill septic patients with and without SLE using univariable and multivariable conditional Cox regression analyses. We found that SLE was independently associated with a high 5-year mortality rate in critically ill septic patients after adjusting relevant variables (HR 1.47, 95% CI 1.22–1.77), and the impact of SLE was consistent using distinct follow-up periods, including 6-month mortality and 1-year mortality (Supplemental Table 3 and 4). In addition to SLE, older age (HR 1.02, 95% CI 1.02–1.02), hospitalised infection within 3 months (HR 1.33, 95% CI 1.12–1.59), and CCI > 3 (HR 2.23, 95% CI 1.97–2.53; CCI = 0–3 as the reference) were associated with 5-year mortality. In detail, the presence of cerebrovascular disease, renal disease, liver disease, tumour, and metastatic tumour were main comorbidities associated with 5-year mortality (Supplemental Table 2). With regard to medications, we found that receiving prednisolone equivalent dose higher than 5 mg/day (HR 1.55, 95% CI 1.27–1.90), methotrexate (HR 2.19, 95% CI 1.61–2.99), and immunosuppressants (HR 1.45, 95% CI 1.22–1.74) were independent risks for mortality (Table 3). Collectively, these data demonstrated that SLE was independently associated with high long-term mortality and identified factors associated with 5-year mortality in critically ill septic patients.

**Table 3:** Crude and adjusted hazard ratios for the association between variable and the risk for 5-year mortality among 2565 enrolled subjects including SLE and matched non-SLE control subjects

	Univariable	Model-1	Model-2	Model-3	Model-4
	Crude Hrs	Adjusted Hrs	Adjusted Hrs	Adjusted Hrs	Adjusted Hrs
**SLE** (non-SLE as reference)	2.27 (2.01–2.57)	2.20 (1.94–2.49)	2.21 (1.95–2.50)	2.01 (1.77–2.27)	1.47 (1.22–1.77)
**Age,** (per 1 year increment)	1.03 (1.02–1.03)	1.03 (1.02–1.03)	1.03 (1.02–1.03)	1.02 (1.01–1.02)	1.02 (1.02–1.02)
**Gender** (male)	1.38 (1.19–1.61)	1.24 (1.07–1.45)	1.23 (1.06–1.44)	1.14 (0.98–1.33)	1.11 (0.95–1.30)
**Urbanisation levels**					
Urban	Ref.		Ref.	Ref.	Ref.
Suburban	1.01 (0.88–1.17)		1.06 (0.92–1.22)	1.09 (0.94–1.25)	1.11 (0.96–1.28)
Rural	0.97 (0.83–1.14)		0.89 (0.76–1.05)	0.92 (0.78–1.07)	0.94 (0.80–1.11)
**Low insured income**^a^	1.01 (0.90–1.13)		1.09 (0.97–1.22)	1.06 (0.95–1.19)	1.08 (0.97–1.21)
**CCI > 3 (0-3 as reference)**	3.06 (2.72–3.43)			2.27 (2.01–2.57)	2.23 (1.97–2.53)
**Recent hospitalised infection**^b^	2.04 (1.75–2.38)			1.35 (1.13–1.60)	1.33 (1.12–1.59)
**Medications**					
**Glucocorticoid dosage group**^c^					
0 mg/day	Ref.				Ref.
0-5 mg/day	1.62 (1.31–2.00)				1.18 (0.95–1.46)
≥ 5 mg/day	2.63 (2.17–3.18)				1.55 (1.27–1.90)
**DMARD**					
Methotrexate	2.55 (1.91–3.39)				2.19 (1.61–2.99)
Sulfasalazine	1.68 (1.03–2.75)				0.84 (0.50–1.42)
Hydroxychloroquine	2.04 (1.78–2.35)				0.98 (0.80–1.19)
Immunosuppressants^d^	2.14 (1.87–2.45)				1.45 (1.22–1.74)

^a^Insured income lower than median income (21,900 New Taiwan dollars). ^b^Within 3 months prior to index admission. ^c^Prednisolone equivalent. ^d^Cyclophosphamide, azathioprine, cyclosporine, mycophenolate, and mycophenolic. *Abbreviations*: *SLE* Systemic lupus erythematosus, *CCI* Charlson comorbidity index, *DMARD* Disease-modifying antirheumatic drug

## Discussion

In this population-based study, we addressed the impact of SLE on the 5-year survival in critically ill septic patients using a case-control design. We found that SLE was associated with high 5-year mortality in patients with sepsis requiring ICU admission and mechanical ventilation. In addition to SLE, older age, higher number of comorbidities, and usage of glucocorticoid (≥ 5 mg/day prednisolone equivalent), methotrexate as well as immunosuppressants were also independent risks for mortality in critically ill septic patients. These findings demonstrated the prolonged impacts of SLE on septic survival and highlight the need for vigilance and risk stratification in lupus patients discharged from ICU for sepsis.

The survival of patients with SLE is improving in the past 4 decades. Yen et al., using a nationwide claim database in the USA, reported that the age-specific SMR for SLE decreased from 0.45 (95% CI, 0.42 to 0.48) per 100,000 person-years in 1968 to 0.34 (95% CI, 0.32 to 0.36) per 100,000 person-years in 2013 [1]. However, Tselios et al. recently investigated the cause- and age-specific SMR among lupus patients in Ontario over a four-decade study period (1971–2013) and found that infection (24.5%) was the leading cause of mortality, followed by atherosclerosis (15.7%), active lupus (13.3%), and malignancy in patients with SLE (9.6%) [2]. In detail, they found that although infection-specific SMR decreased steadily by decades, but the infection-specific SMR was extremely high (30.2, 95% CI 14.4–46.0) in lupus patients aged 19–39 compared with the relatively low SMR (3.5; 95% CI 2.5–4.5) among those older or equal to 40 years [2]. Similarly, hospitalisation for serious infections among lupus patients in the USA increases steadily between 1996 and 2011, with nearly 12 times higher than those in non-SLE populations [20], and sepsis has been reported to account for nearly 80% of aetiologies for ICU admission in Thailand among patients with SLE [21]. These evidence highlight the essential need for investigating the association between SLE and sepsis relevant mortality.

Despite of a steady decrease in ICU mortality, a number of studies including our studies have found high post-ICU mortality in the past two decades [9, 12, 22]. Therefore, the long-term outcome, instead of ICU/hospital-mortality, of critically ill patients is currently one of the leading research priorities in critical care medicine, particularly among those with sepsis, given the increasing evidence have shown the prolonged immunologic and metabolic sequelae of sepsis [10, 13, 14]. van Vught et al., employing paired analyses of the whole transcriptome in leucocytes among septic patients with and without secondary infection, found impaired gluconeogenesis and glycolysis in septic patients with a secondary infection [11]. In addition to secondary infection, the post-sepsis altered immunologic and metabolic function may have systemic impact in patients with sepsis as shown by one Taiwanese population-based study that sepsis survivors had an increased risk of all-cause mortality (HR 2.18; 95% CI 2.14–2.22) and major adverse cardiovascular events at 1 year after discharge (HR, 1.37; 95% CI, 1.34–1.41) [12]. Given that the immune system plays a substantial role in the long-term outcome after the septic episode, there is a crucial need to explore the long-term outcome in critically ill septic patients with autoimmune diseases including SLE [13]. Indeed, few studies focused on investigating long-term outcome in critically ill septic patients with SLE. One Taiwanese single-hospital study, investigating 240 lupus patients with bacteremia between 2000 and 2005, reported the 30-day and 1-year mortality rate was 24% and 33%, respectively [23]. Larcher et al., investigating 525 critically ill patients with systemic rheumatic disease including 109 lupus patients, reported that ICU-, hospital-, and 1-year-mortality rates were 23.8%, 30.5%, and 37.7%, respectively [8]. Due to the distinct young population and the markedly changed survival of SLE in recent years, there is a crucial need to investigate the long-term outcome of critically ill patients with SLE. In the present study, we used data mainly in female whose age was approximately 50 years to provide the real-world 5-year mortality data among critically ill septic patients with and without SLE and to show the independent mortality impact of SLE in critically ill septic patients. Collectively, these findings indicate vigilance for lupus patient discharged from ICU for sepsis, and more studies are warranted to elucidate underlying biological mechanisms.

The impacts of SLE on critically ill septic patients may result from an altered immunological and metabolic response in the recovery from sepsis. Shi et al., conducting RNA-seq of monocytes in 9 lupus patients and matched control subjects, showed evidence of chronic endotoxin exposure and differentially expressed type I interferon (IFN) genes in lupus patients [24]. Indeed, type I IFN has been implicated with a wide range of infectious diseases, including bacterial, mycobacterial, and viral infection [25–28]. Yang et al., using a mouse sepsis model with cecal ligation and puncture (CLP), recently reported that type I IFN exerted the disseminated intravascular coagulation in bacterial infection through amplifying the release of high-mobility group box 1 (HMGB1) into the extracellular space [25]. Notably, HMGB1 was recently identified to be associated with a prolonged and impaired cognitive function in patient survived from a critical illness [29]. Therefore, type I IFN and HMGB1 has been seen as the potential therapeutic target in sepsis [30, 31]. Type I IFN has also been implicated with a dysregulated inflammation in mycobacterial infection, and increasing studies including our previous study have found that pro-inflammatory mediators including IL-1β and type I IFN strengthen the eicosanoid pathway, which in turn modulates death patterns of infected cells in mycobacterial infection [26, 32, 33]. Notably, Clayton et al. revealed an overlapped transcriptomic signature, mainly type I IFN-associated signalling pathway, between patients with tuberculosis and SLE [34]. Two studies further revealed that impaired type I IFN immunity, including autoantibodies against type I IFN and inborn errors of type I IFN immunity, may lead to severe coronavirus disease 19 (COVID-19) infection [27, 28].

Surprisingly, most of the studies with regard to the survival in lupus patients after sepsis/infection mainly investigate patients with SLE and healthy controls in the general population, and few studies have comparable septic controls to specify the independent impact of SLE on the long-term outcome of sepsis. Kedves et al. recently conducted a population-based claim database in Hungary with age- and sex-matched health controls to explore the long-term impact of patients with SLE [4]. They found an increased adjHR (2.17, 95% CI 1.94–2.44) for all-cause mortality in patients with SLE compared with healthy controls and reported higher infection-related deaths in lupus patients than those in healthy control subjects [4]. Similarly, one population-based study conducted in southern Sweden also reported a higher long-term mortality rate in patients with SLE compared with the mortality rate in the general population [6]. One recently published study using the 2010–2015 French SLE cohort reported that 1068 lupus patients with septic shock had higher 1-year mortality than lupus patients without septic shock [35]. These evidence highlight the crucial need for comparable non-SLE septic controls with similar age as well as sex to clarify the independent impact of SLE on the long-term outcome of sepsis.

In the present study, we found that usage of glucocorticoid, methotrexate, and immunosuppressants, but not hydroxychloroquine, as well as more comorbidity, including chronic kidney disease, were associated with high 5-year mortality. Intriguingly, previous studies including our recently published study have shown that the use of hydroxychloroquine tended to inversely be associated with incident infectious disease, particularly malaria and pneumocystis pneumonia (PCP), among patients with SLE [36, 37]. We further conducted analyses focusing on the usage of hydroxychloroquine among the 513 enrolled critically ill septic patients with sepsis; however, we did not observe the protective effect of hydroxychloroquine on post-septic 5-year mortality (Supplemental Table 5–7). We postulated the relatively low incidence of hydroxychloroquine-protected diseases in Taiwan and attributed mortality might at least partly explain the lack of association between the use of hydroxychloroquine and 5-year mortality in the present study.

We applied the sepsis-3 definition, using the SOFA score to identify patients with sepsis, to define sepsis in the present study [15]. Compared with the sepsis-3 definition, the sepsis-2 definition, using a requisite minimum of two systemic inflammatory response syndrome criteria, might not be not fully accurate to identify patients with sepsis [38]. As shown by Kaukonen et al., one in eight critically ill patients admitted to an ICU for infection with new organ failure is estimated not to meet the sepsis-2 definition, and these patients exhibit significant mortality and morbidity [38]. Therefore, the Sepsis-3 definition is increasingly used to identify patients with sepsis in recent studies including our recently published study to address factors for sepsis in patients receiving tumour necrosis factor inhibitors for immune-mediated inflammatory diseases [17, 39, 40].

There are limitations to this study. First, we used ICD coding to define patient with sepsis, and sepsis could potentially be overestimated. However, we used a stringent definition by restricting septic patients admitted to the ICU and received mechanical ventilation. Therefore, we think we might underestimate, instead of overestimate, the critically ill septic patients under such a stringent definition. Second, the lack of data regarding disease activity in claim data is a limitation; however, the comprehensive information regarding medications should at least partly reflect the disease activity of SLE. Similarly, the cause of death cannot be delineated in NHIRD. Third, the concern for the accuracy of the SLE diagnosis in claims data, but the diagnosis of SLE in Taiwan was validated by at least two qualified rheumatologists through checking clinical data for the certificate of catastrophic illness. Fourth, more studies are warranted to validate our findings in other populations.

## Conclusions

In conclusion, the overall survival in patients with SLE is changing, and sepsis is currently the leading cause-of-death, particularly among lupus patients with younger age. Therefore, there is a crucial need for studies with comparable septic controls to delineate the long-term mortality impact of SLE in critically ill septic patients. In this population-based case-control study, we identified a marked impact of SLE on the 5-year mortality among patients with sepsis requiring ICU admission and mechanical ventilation. We also found that an older age, higher number of comorbidities, and usage of glucocorticoid, methotrexate as well as immunosuppressants contributed to increased mortality in critically ill septic patients. These findings provide evidence for mortality risk stratification in lupus patients who survived from sepsis, and future studies are required to clarify underlying mechanisms.

## Supplementary Information

**Additional file 1: Supplemental table 1**. Patients with sepsis categorised by presence of SLE, admission to ICU, and requirment of mechanical ventilator. **Supplemental table 2**. Crude and adjusted hazard ratios for the association between variable and the risk for 5-year mortality among 2,565 enrolled subjects including SLE and matched non-SLE control subjects. **Supplemental table 3**. Crude and adjusted hazard ratios for the association between variable and the risk for 6-month mortality among 2,565 enrolled subjects including SLE and matched non-SLE control subjects. **Supplemental table 4** . Crude and adjusted hazard ratios for the association between variable and the risk for 1-year mortality among 2,565 enrolled subjects including SLE and matched non-SLE control subjects. **Supplemental table 5**. Demographic data and clinical characteristics among 513 critically ill lupus patients categorised by the use of hydroxychloroquine. **Supplemental table 6**. Mortality in the 513 critically ill lupus patients who survived from sepsis with and without the use of hydroxychloroquine. **Supplemental table 7**. Crude and adjusted hazard ratios for the association between variable and the risk for 5-year mortality among 513 critically ill lupus patients who survived from sepsis.

## Data Availability

The authors confirm that the data supporting the findings of the present study are available within the manuscript and the supplemental data.
