# Supplementary material for: Impact of systemic lupus erythematosus on the 5-year survival of critically ill septic patients
Source: Arthritis Res Ther. 2021 Oct 21;23:264. doi: 10.1186/s13075-021-02649-x (PMC8528659; doi:10.1186/s13075-021-02649-x)
Supplement: Supplementary file 1 — Additional file 1: Supplemental table 1. Patients with sepsis categorised by presence of SLE, admission to ICU, and requirment of mechanical ventilator. Supplemental table 2. Crude and adjusted hazard ratios for the association between variable and the risk for 5-year mortality among 2,565 enrolled subjects including SLE and matched non-SLE control subjects. Supplemental table 3. Crude and adjusted hazard ratios for the association between variable and the risk for 6-month mortality among 2,565 enrolled subjects including SLE and matched non-SLE control subjects. Supplemental table 4 . Crude and adjusted hazard ratios for the association between variable and the risk for 1-year mortality among 2,565 enrolled subjects including SLE and matched non-SLE control subjects. Supplemental table 5. Demographic data and clinical characteristics among 513 critically ill lupus patients categorised by the use of hydroxychloroquine. Supplemental table 6. Mortality in the 513 critically ill lupus patients who survived from sepsis with and without the use of hydroxychloroquine. Supplemental table 7. Crude and adjusted hazard ratios for the association between variable and the risk for 5-year mortality among 513 critically ill lupus patients who survived from sepsis. [file 13075_2021_2649_MOESM1_ESM.pdf]

Supplemental table 1. Patients with sepsis categorised by presence of SLE, admission to ICU, and requirment of mechanical ventilator

	SLE	Non-SLE
**All of patients with sepsis**	3198	822,358
**ICU (-), Ventilator (-)**	1,466 (45.8%)	321,076 (39.0%)
**ICU (+), Ventilator (-)**	395 (12.4%)	151,899 (18.5%)
**ICU (-), Ventilator (+)**	102 (3.2%)	31,699 (3.9%)
**ICU (+), Ventilator (+)**	1,235 (38.6%)	317,684 (38.6%)

Abbreviations: SLE, systemic lupus erythematosus; ICU, intensive care unit.

.

Supplemental table 2. Crude and adjusted hazard ratios for the association between variable and the risk for 5-year mortality among 2,565 enrolled subjects including SLE and matched non-SLE control subjects

	**Univariable crude HRs**	**p-value**	**Model-1 adjusted HRs**	**Model-2 adjusted HRs**	**Model-3 adjusted HRs**	**Model-4 adjusted HRs**
**SLE** (Non-SLE as reference)	2.27 (2.01–2.57)	<0.001	2.20 (1.94–2.49)	2.16 (1.90–2.45)	2.35 (2.03–2.71)	1.78 (1.46–2.17)
**Age,** (per 1 year increment)	1.03 (1.02–1.03)	<0.001	1.03 (1.02–1.03)	1.02 (1.02–1.03)	1.02 (1.02–1.03)	1.02 (1.02–1.03)
**Gender** (Male)	1.38 (1.19–1.61)	<0.001	1.24 (1.07–1.45)	1.20 (1.03–1.39)	1.14 (0.98–1.33)	1.13 (0.97–1.32)
**Urbanisation levels**						
Urban	Ref.			Ref.	Ref.	Ref.
Suburban	1.01 (0.88–1.17)	0.851		1.07 (0.92–1.23)	1.14 (0.99–1.32)	1.16 (1.01–1.34)
Rural	0.97 (0.83–1.14)	0.731		0.89 (0.76–1.04)	1.00 (0.85–1.17)	1.01 (0.86–1.19)
**Low insured income**[a]	1.01 (0.90–1.13)	0.868		1.09 (0.97–1.22)	1.11 (0.99–1.25)	1.13 (1.001–1.26)
**Recent hospitalised infection**[b]	2.04 (1.75–2.38)	<0.001		1.50 (1.26–1.79)	1.19 (0.99–1.42)	1.18 (0.99–1.42)
**Comorbidities**						
Myocardial Infarction	1.30 (0.93–1.81)	0.124			1.20 (0.85–1.69)	1.22 (0.86–1.71)
Congestive heart failure	1.46 (1.25–1.70)	<0.001			1.10 (0.93–1.30)	1.10 (0.93–1.30)
Peripheral vascular disease	1.34 (0.91–1.98)	0.140			1.19 (0.80–1.77)	1.23 (0.83–1.83)
Cerebrovascular disease	1.46 (1.27–1.67)	<0.001			1.18 (1.02–1.37)	1.20 (1.04–1.39)
Dementia	1.91 (1.43–2.56)	<0.001			1.08 (0.79–1.48)	1.09 (0.80–1.49)
Chronic pulmonary disease	1.47 (1.27–1.69)	<0.001			1.12 (0.96–1.30)	1.11 (0.95–1.29)
Rheumatic disease[c]	2.29 (2.02–2.59)	<0.001				
Peptic ulcer disease	1.67 (1.46–1.92)	<0.001			1.05 (0.91–1.22)	1.05 (0.91–1.21)

Diabetes mellitus without end-organ damage	1.17 (1.03–1.33)	0.014	1.00 (0.86–1.16)	1.01 (0.87–1.18)
DM with end-organ damage	1.42 (1.18–1.70)	<0.001	1.15 (0.93–1.43)	1.18 (0.95–1.46)
Hemiplegia	0.94 (0.60–1.47)	0.777		
Renal disease	1.99 (1.74–2.28)	<0.001	1.49 (1.28–1.74)	1.48 (1.27–1.72)
Tumour	3.22 (2.81–3.69)	<0.001	2.42 (2.04–2.87)	2.31 (1.94–2.75)
Liver disease	2.76 (2.13–3.58)	<0.001	2.64 (1.95–3.56)	2.94 (2.17–3.98)
Metastatic solid tumour	4.03 (3.35–4.85)	<0.001	2.71 (2.14–3.43)	2.55 (2.01–3.22)
Human immunodeficiency virus	1.22 (0.46–3.25)	0.696		
**Medications**				
**Glucocorticoid dosage group**[d]				
0 mg/day	Ref.			Ref.
0-5 mg/day	1.62 (1.31–2.00)	<0.001		1.11 (0.90–1.38)
≥5 mg/day	2.63 (2.17–3.18)	<0.001		1.38 (1.12–1.69)
**DMARD**				
Methotrexate	2.55 (1.91–3.39)	<0.001		2.11 (1.55–2.86)
Sulfasalazine	1.68 (1.03–2.75)	0.039		0.85 (0.51–1.43)
Hydroxychloroquine	2.04 (1.78–2.35)	<0.001		1.05 (0.86–1.28)
Immunosuppressants[e]	2.14 (1.87–2.45)	<0.001		1.38 (1.16–1.65)

[a]Insured income lower than median income (21,900 New Taiwan dollars). [b]Within 3 months prior to index admission.[c]Not included in multivariable due to a high variance inflation factor (16.1). [d]Prednisolone equivalent. [e]Cyclophosphamide, azathioprine, cyclosporine, mycophenolate, and mycophenolic. Abbreviations: SLE, systemic lupus erythematosus; CCI, Charlson comorbidity index; DMARD, disease-modifying antirheumatic drug.

Supplemental table 3. Crude and adjusted hazard ratios for the association between variable and the risk for 6-month mortality among 2,565 enrolled subjects including SLE and matched non-SLE control subjects

	Univariable crude HRs	Model-1 adjusted HRs	Model-2 adjusted HRs	Model-3 adjusted HRs	Model-4 adjusted HRs
**SLE** (Non-SLE as reference)	2.37 (2.04–2.74)	2.33 (2.01–2.71)	2.31 (1.99–2.68)	2.13 (1.83–2.48)	1.46 (1.17–1.84)
**Age,** (per 1 year increment)	1.02 (1.01–1.02)	1.02 (1.01–1.02)	1.02 (1.01–1.02)	1.01 (1.004–1.01)	1.01 (1.01–1.02)
**Gender** (Male)	1.18 (0.97–1.43)	1.09 (0.90–1.33)	1.09 (0.90–1.32)	1.00 (0.82–1.22)	0.97 (0.80–1.19)
**Urbanisation levels**					
Urban	Ref.		Ref.	Ref.	Ref.
Suburban	0.98 (0.83–1.17)		1.05 (0.89–1.25)	1.09 (0.91–1.29)	1.12 (0.94–1.34)
Rural	0.87 (0.72–1.05)		0.87 (0.72–1.06)	0.90 (0.74–1.10)	0.93 (0.77–1.14)
**Low insured income**[a]	0.89 (0.77–1.02)		0.96 (0.83–1.10)	0.93 (0.81–1.08)	0.95 (0.82–1.09)
**CCI >3 (0-3 as reference)**	2.61 (2.27–3.01)			2.17 (1.87–2.53)	2.12 (1.82–2.47)
**Recent hospitalised infection**[b]	1.87 (1.55–2.26)			1.34 (1.09–1.66)	1.32 (1.07–1.64)
**Medications**					
**Glucocorticoid dosage group**[c]					
0 mg/day	Ref.				Ref.
0-5 mg/day	1.53 (1.17–2.01)				1.19 (0.90–1.57)
≥5 mg/day	2.65 (2.08–3.38)				1.54 (1.18–1.99)
**DMARD**					
Methotrexate	2.87 (2.10–3.92)				2.28 (1.63–3.20)
Sulfasalazine	1.63 (0.90–2.95)				0.82 (0.44–1.53)
Hydroxychloroquine	2.12 (1.80–2.50)				0.95 (0.75–1.20)
Immunosuppressants[d]	2.52 (2.15–2.95)				1.70 (1.37–2.10)

[a]Insured income lower than median income (21,900 New Taiwan dollars). [b]Within 3 months prior to index admission. [c]Prednisolone equivalent. [d]Cyclophosphamide, azathioprine, cyclosporine, mycophenolate, and mycophenolic. Abbreviations: SLE, systemic lupus erythematosus; CCI, Charlson comorbidity index; DMARD, disease-modifying antirheumatic drug.

Supplemental table 4. Crude and adjusted hazard ratios for the association between variable and the risk for 1-year mortality among 2,565 enrolled subjects including SLE and matched non-SLE control subjects

	**Univariable crude HRs**	**Model-1 adjusted HRs**	**Model-2 adjusted HRs**	**Model-3 adjusted HRs**	**Model-4 adjusted HRs**
**SLE** (Non-SLE as reference)	2.30 (2.00–2.65)	2.26 (1.96–2.60)	2.24 (1.95–2.58)	2.07 (1.79–2.38)	1.49 (1.20–1.84)
**Age,** (per 1 year increment)	1.02 (1.02–1.02)	1.02 (1.02–1.02)	1.02 (1.02–1.02)	1.01 (1.01–1.02)	1.02 (1.01–1.02)
**Gender** (Male)	1.21 (1.01–1.45)	1.11 (0.93–1.33)	1.10 (0.92–1.32)	1.02 (0.85–1.22)	0.99 (0.82–1.18)
**Urbanisation levels**					
Urban	Ref.		Ref.	Ref.	Ref.
Suburban	1.00 (0.85–1.17)		1.06 (0.90–1.25)	1.09 (0.93–1.29)	1.13 (0.96–1.32)
Rural	0.90 (0.75–1.08)		0.87 (0.73–1.05)	0.90 (0.75–1.08)	0.93 (0.77–1.12)
**Low insured income**[a]	0.92 (0.81–1.05)		0.99 (0.87–1.13)	0.96 (0.85–1.10)	0.98 (0.86–1.12)
**CCI >3 (0-3 as reference)**	2.70 (2.37–3.08)			2.15 (1.87–2.48)	2.11 (1.83–2.43)
**Recent hospitalised infection**[b]	1.93 (1.62–2.30)			1.36 (1.12–1.66)	1.35 (1.11–1.65)
**Medications**					
**Glucocorticoid dosage group**[c]					
0 mg/day	Ref.				Ref.
0-5 mg/day	1.48 (1.15–1.89)				1.12 (0.87–1.44)
≥5 mg/day	2.54 (2.03–3.16)				1.49 (1.18–1.89)
**DMARD**					
Methotrexate	2.66 (1.96–3.61)				2.23 (1.60–3.11)
Sulfasalazine	1.57 (0.89–2.78)				0.80 (0.44–1.46)
Hydroxychloroquine	2.04 (1.74–2.38)				0.93 (0.75–1.17)
Immunosuppressants[d]	2.32 (1.99–2.69)				1.58 (1.30–1.93)

[a]Insured income lower than median income (21,900 New Taiwan dollars). [b]Within 3 months prior to index admission. [c]Prednisolone equivalent. [d]Cyclophosphamide, azathioprine, cyclosporine, mycophenolate, and mycophenolic. Abbreviations: SLE, systemic lupus erythematosus; CCI, Charlson comorbidity index; DMARD, disease-modifying antirheumatic drug.

Supplemental table 5. Demographic data and clinical characteristics among 513 critically ill lupus patients categorised by the use of hydroxychloroquine

	Hydroxychloroquine (-), n=182	Hydroxychloroquine (+), n=331	p-value
**Age, years**	52.2±16.5	46.9±15.9	<0.01
**Gender**			0.04
Female	149 (81.9)	293 (88.5)	
Male	33 (18.1)	38 (11.5)	
**Urbanisation levels**			0.16
Urban	65 (35.7)	99 (29.9)	
Suburban	80 (44.0)	141 (42.6)	
Rural	37 (20.3)	91 (27.5)	
**Low insured income**[a]	89 (48.9)	154 (46.5)	0.61
**CCI,** mean ± SD	3.1±2.0	2.7±1.5	0.02
**CCI,** group			<0.01
0-3	113 (62.1)	250 (75.5)	
>3	69 (37.9)	81 (24.5)	
**Recent hospitalised infection**	24 (13.2)	54 (16.3)	0.35
**Medications**			
**Glucocorticoid use**	175 (96.2)	331 (100)	<0.01
**Glucocorticoid dosage, mg/day**[c]	52.6±92.2	45.3±80.9	0.37
**Glucocorticoid dosage group**[c]			<0.01
0 mg/day	7 (3.8)	0 (0.0)	
0-5 mg/day	13 (7.1)	14 (4.2)	
≥5 mg/day	162 (89)	317 (95.8)	
**DMARD**			
Methotrexate	4 (2.2)	16 (4.8)	0.14
Immunosuppressants[d]	83 (45.6)	211 (63.7)	<0.01

[a]Insured income lower than median income (21,900 New Taiwan dollars). [b]Within 3 months prior to index admission [c]Prednisolone equivalent. [d]Cyclophosphamide, azathioprine, cyclosporine, mycophenolate, and mycophenolic.
Abbreviations: CCI, Charlson comorbidity index; DMARD, disease-modifying antirheumatic drug.

Supplemental table 6. Mortality in the 513 critically ill lupus patients who survived from sepsis with and without the use of hydroxychloroquine

	Total	Event (%)	Total person-years	Incidence Rate (/10^5 years)	Crude IRR (95%CI)	*p* value
**6-month mortality**						
**Hydroxychloroquine (-)**	182	93 (51.10)	55	169,806	1	
**Hydroxychloroquine (+)**	331	171 (51.66)	99	172,103	1.01 (0.79-1.30)	0.917
**1-year mortality**						
**Hydroxychloroquine (-)**	182	104 (57.14)	95	109,385	1	
**Hydroxychloroquine (+)**	331	188 (56.80)	172	109,424	1.00 (0.79-1.27)	0.998
**5-year mortality**						
**Hydroxychloroquine (-)**	182	131 (71.98)	325	40,347	1	
**Hydroxychloroquine (+)**	331	232 (70.09)	618	37,554	0.93 (0.75-1.15)	0.512

Abbreviations: IRR, Incidence rate ratio; CI, confidence interval.

Supplemental table 7. Crude and adjusted hazard ratios for the association between variable and the risk for 5-year mortality among 513 critically ill lupus patients who survived from sepsis

	Univariable		Multivariable	
	**HR (95% CI)**	***p* value**	**HR (95%CI)**	***p* value**
**Age,** (per 1 year increment)	1.01 (1.01–1.02)	0.001	1.01 (1.01–1.02)	0.001
**Gender** (Male)	0.88 (0.65–1.19)	0.411	0.77 (0.57–1.06)	0.108
**Urbanisation levels**				
Urban	Reference		Reference	
Suburban	1.00 (0.79–1.27)	0.986	1.04 (0.82–1.33)	0.747
Rural	0.99 (0.75–1.30)	0.944	0.98 (0.74–1.30)	0.882
**Low insured income**[a]	0.96 (0.78–1.18)	0.706	1.00 (0.81–1.23)	0.989
CCI, 0-3	Reference		Reference	
CCI >3	1.28 (1.03–1.60)	0.025	1.19 (0.95–1.50)	0.131
**Recent hospitalised infection**[b]	1.44 (1.10–1.88)	0.008	1.32 (0.98–1.78)	0.065
**Medications**				
**Glucocorticoid dosage group**[c]				
0 mg/day	Reference		Reference	
0-5 mg/day	1.08 (0.36–3.19)	0.897	1.08 (0.36–3.25)	0.889
≥5 mg/day	1.46 (0.55–3.91)	0.452	1.72 (0.62–4.73)	0.295
**DMARD**				
Methotrexate	1.07 (0.63–1.82)	0.811	1.23 (0.71–2.12)	0.455
Hydroxychloroquine	0.96 (0.78–1.19)	0.727	0.96 (0.76–1.20)	0.704
Immunosuppressants[d]	1.01 (0.82–1.24)	0.932	1.16 (0.92–1.47)	0.204

[a]Insured income lower than median income (21,900 New Taiwan dollars). [b]Within 3 months prior to index admission.

[c]Prednisolone equivalent. [d]Cyclophosphamide, azathioprine, cyclosporine, mycophenolate, and mycophenolic.

Abbreviations: CCI, Charlson comorbidity index; DMARD, disease-modifying antirheumatic drug.
